# Selective α_3_β_4_ Nicotinic Acetylcholine Receptor Ligand as a Potential Tracer for Drug Addiction

**DOI:** 10.3390/ijms24043614

**Published:** 2023-02-10

**Authors:** Apinan Kanasuwan, Winnie Deuther-Conrad, Sumet Chongruchiroj, Jiradanai Sarasamkan, Chanisa Chotipanich, Opa Vajragupta, Kuntarat Arunrungvichian

**Affiliations:** 1 Department of Pharmaceutical Chemistry, Faculty of Pharmacy, Mahidol University, 447 Sri-Ayutthaya Rd., Bangkok 10400, Thailand; 2 National Cyclotron and PET Centre, Chulabhorn Hospital, Chulabhorn Royal Academy, 906 Kamphaengphet 6 Rd., Bangkok 10210, Thailand; 3 Department of Neuroradiopharmaceuticals, Institute of Radiopharmaceutical Cancer Research, Helmholtz-Zentrum Dresden-Rossendorf, Permoserstraße 15, 04318 Leipzig, Germany; 4 Department of Microbiology, Faculty of Pharmacy, Mahidol University, 447 Sri-Ayutthaya Rd., Bangkok 10400, Thailand; 5 Department of Radiology, Faculty of Medicine, Khon Kaen University, 123 Mittraphap Rd., Khon Kaen 40002, Thailand; 6 Molecular Probes for Imaging Research Network, Faculty of Pharmaceutical Sciences, Chulalongkorn University, 254 Phayathai Rd., Bangkok 10330, Thailand

**Keywords:** α_3_β_4_ Nicotinic acetylcholine receptor, quinuclidine, triazole, drug-seeking behavior monitoring, drug addiction

## Abstract

α_3_β_4_ Nicotinic acetylcholine receptor (nAChR) has been recognized as an emerging biomarker for the early detection of drug addiction. Herein, α_3_β_4_ nAChR ligands were designed and synthesized to improve the binding affinity and selectivity of two lead compounds, **(*S*)**-**QND8** and **(*S*)**-**T2**, for the development of an α_3_β_4_ nAChR tracer. The structural modification was achieved by retaining the key features and expanding the molecular structure with a benzyloxy group to increase the lipophilicity for blood-brain barrier penetration and to extend the ligand-receptor interaction. The preserved key features are a fluorine atom for radiotracer development and a *p*-hydroxyl motif for ligand-receptor binding affinity. Four (*R*)- and (*S*)-quinuclidine-triazole (**AK1**-**AK4**) were synthesized and the binding affinity, together with selectivity to α_3_β_4_ nAChR subtype, were determined by competitive radioligand binding assay using [^3^H]epibatidine as a radioligand. Among all modified compounds, **AK3** showed the highest binding affinity and selectivity to α_3_β_4_ nAChR with a *K_i_* value of 3.18 nM, comparable to **(*S*)**-**QND8** and **(*S*)**-**T2** and 3069-fold higher affinity to α_3_β_4_ nAChR in comparison to α_7_ nAChR. The α_3_β_4_ nAChR selectivity of **AK3** was considerably higher than those of **(*S*)**-**QND8** (11.8-fold) and **(*S*)**-**T2** (294-fold). **AK3** was shown to be a promising α_3_β_4_ nAChR tracer for further development as a radiotracer for drug addiction.

## 1. Introduction

Nicotinic acetylcholine receptors (nAChRs) are ligand-gated ion channel receptors in the Cys-loop superfamily, which are expressed in the central nervous system (CNS) and in the peripheral nervous system (PNS) [1,2,3]. The subunits of nAChRs can be alpha (α_2_–α_10_) and beta (β_2_–β_4_) subunits surrounding an ion pore. The nAChR subtypes can be divided into two classes, according to subunit assembly: homopentameric receptors such as α_7_ and α_9_ and heteropentameric receptors such as α_3_β_4_ and α_4_β_2_ [3]. The different subunit combinations bring about the distinct pharmacological profile of nAChR [1]. For example, the α_7_ and α_4_β_2_ nAChR subtypes, which are expressed at high levels in the brain, are involved in neurological disorders (Alzheimer’s disease, schizophrenia, Parkinson’s disease, attention deficit hyperactivity disorder (ADHD), and inflammation [4,5,6,7,8,9,10]), whereas the α_3_β_4_ nAChR is involved in depression and drug addiction [11,12,13].

Drug addiction, particularly nicotine addiction, is a worldwide epidemic that affects the lives of both smokers and non-smokers. The reward and reinforcement effects, through the mesolimbic pathway, and the aversive effects of withdrawal, via the medial habenula-interpeduncular (MHb-IPN) pathway, are involved in maintaining nicotine use [14,15,16]. Examples of subunits in nAChR subtypes that are mainly expressed in the mesolimbic pathway are α_4_, α_6_, α_7,_ and β_2_ subunits [17], whereas α_3_, α_5,_ and β_4_ subunits are in the MHb-IPN circuit [13,18,19]. Human genetic studies showed that gene clusters expressing α_3_, α_5,_ and β_4_ subunits were associated with tobacco dependence and higher numbers of cigarettes smoked per day [20,21,22]. The expression of α_3_, α_5,_ and β_4_ nAChR subunits in the MHb-IPN circuit has drawn attention to the study of both α_3_β_4_* and α_5_* nAChRs in nicotine aversion. However, some evidence supports the role of α_4_β_2_α_5_* nAChR in regulating glutamate transmission [23] and that of α_3_β_4_* nAChR in regulating acetylcholine release in the MHb-IPN pathway [24,25,26].

**Figure 1:**
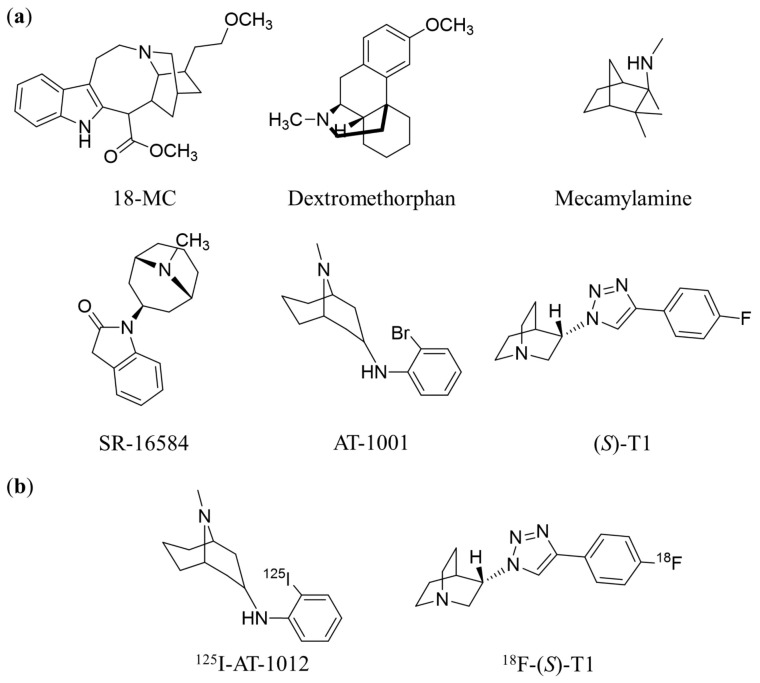
Structures of (**a**) α_3_β_4_ nAChR ligands and (**b**) radio-imaging agents.

The α_3_β_4_ nAChR expressed in the MHb-IPN circuit [14,27,28,29] has been reported to play a role in drug or psychostimulant-seeking behavior including nicotine, morphine, methamphetamine, and alcohol [30]. The α_3_β_4_ nAChR is not only expressed in the interpeduncular nucleus (IPN) and the medial habenula (MHb), but also in the pineal gland, locus coeruleus, and hippocampus [31]. Several compounds are reported to bind with α_3_β_4_ nAChR, including 18-methoxycoronaridine (18-MC) [32], dextromethorphan [33], mecamylamine [34], SR16584 [35], (*S*)-T1 [36] and AT-1001 [37] (Figure 1). However, all of them except (*S*)-T1 and AT-1001 are non-selective ligands, which can bind with other subtypes or other receptors. For example, 18-MC can bind to an opioid receptor [38] and a muscle AChR [39], mecamylamine can antagonize several nAChR subtypes [40] and dextromethorphan can block N-methyl-D-aspartate (NMDA) receptor [41]. The selective α_3_β_4_ nAChR (*S*)-T1 and AT-1001 with *K_i_* below 10 nanomolar were further developed to be α_3_β_4_ nAChR radiotracers by labeling with a radionuclide yielding [^125^I]AT-1012 [42] and (*S*)-[^18^F]T1 [36], respectively. [^125^I]AT-1012 labeled with ^125^I, a gamma-emitter, is a single-photon emission computed tomography (SPECT) tracer, while (*S*)-[^18^F]T1 containing ^18^F, a positron emitter, is a positron emission tomography (PET) imaging agent. The selective α_3_β_4_ nAChR radiotracer is a tool for studying psychostimulant and drug-seeking behavior and drug discovery.

Nowadays, the combined use of a radiotracer and a PET scanner is the most precise imaging technique to detect early-stage of diseases at a molecular level [43]. The scanner quantitatively measures biochemical and physiological processes by using a suitable radiotracer. The radiotracer comprises two parts: a specific tracer to localize a target or disease biomarker and a positron-emitting radionuclide, such as fluorine-18 (^18^F) or carbon-11 (^11^C), to produce gamma radiation via the process of positron annihilation [44]. The development of a radiotracer has three main steps: (i) design and synthesis of non-radioactive reference compound (formerly named cold standard), where the radionuclide in a radiotracer is a non-radioactive isotope; (ii) synthesis of a precursor for radiolabeling; and (iii) radiosynthesis or radiolabeling of a precursor to yield a radiotracer [45]. The starting point for the development of a new radiotracer is the design of a new tracer or search for available non-radioactive reference compounds with good binding affinity at a nanomolar level and high selectivity profile to a target (disease biomarker).

In this study, four compounds (**AK1**-**AK4**) were designed to increase the binding affinity and selectivity to α_3_β_4_ nAChR. The designed structures were synthesized by copper-catalyzed azide-alkyne cycloaddition (CuAAC) and evaluated in silico and in vitro for binding affinity and selectivity. The compound showing the highest affinity and selectivity to α_3_β_4_ nAChR will be selected as a non-radioactive reference compound for further development as an α_3_β_4_ nAChR PET imaging agent for drug addiction.

## 2. Results and Discussion

**Figure 2:**
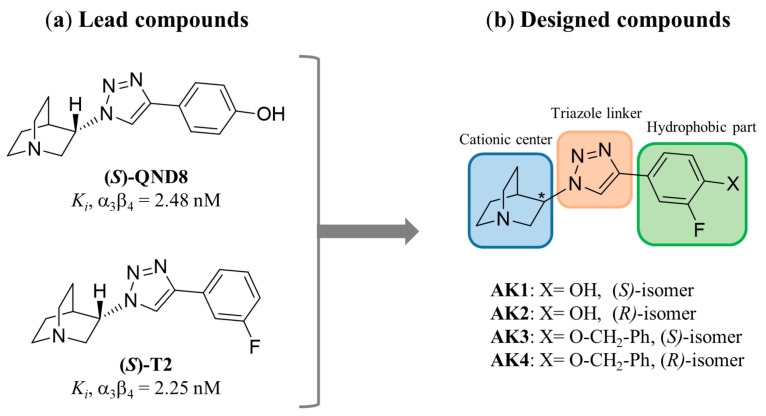
The design strategy of α_3_β_4_ nAChR ligands.

Four quinuclidine-triazole compounds (**AK1**-**AK4**) were modified by merging the structures of two lead compounds, **(*S*)**-**QND8** and **(*S*)**-**T2** [46], followed by single point modification (Figure 2) to improve α_3_β_4_ nAChR binding affinity and selectivity profiles. Two pharmacophoric features, a chiral quinuclidine ring ((*R*) and (*S*)) acting as a cationic center and a triazole linker acting as a hydrogen bond acceptor [47], were kept in the modified structures. The key functional motifs in the hydrophobic part of **(*S*)**-**QND8** and **(*S*)**-**T2** were preserved: a hydroxyl group (-O**H**) for forming a hydrogen bond with the receptor [48] and a fluorine atom for further development as a positron-emitting radionuclide, fluorine-18. Besides retaining the fluorine atom and the hydroxyl group, the single-point modification was made by extending the hydrophobic part with a benzene ring at the hydroxyl function. The benefit of the added benzyloxy is that it can increase the lipophilicity of **(*S*)**-**QND8** and **(*S*)**-**T2** from log P of 1.87 and 2.03, respectively to log P of 4.03 and accordingly enhance brain penetration and subtype selectivity. The hydrophobic area has been reported to mediate nAChR subtype selectivity.

The binding affinities (*K_i_*) of **AK1**-**AK4** and two lead compounds were presented in Table 1. All compounds exhibited binding affinity to α_3_β_4_ nAChR, α_7_ nAChR, and α_4_β_2_ nAChR in the range of 2.28–9760 nM. Comparing (*R*)- and (*S*)-enantiomers, the (*S*)-isomers of both **AK1** and **AK3** preferably bound to α_3_β_4_ nAChR, whereas their corresponding (*R*)-isomers (**AK2** and **AK4**) selectivity bound to α_7_ nAChR. The stereoselective binding of (*S*)-enantiomers to α_3_β_4_ nAChR and (*R*)-enantiomers to α_7_ nAChR agrees with previous reports [46,48]. This might lead to some limitations of this study. The data discussed stereoselectivity came from one core structure, which is a quinuclidine ring.

The binding affinities of new (*S*)-enantiomers (**AK1** and **AK3**) to α_3_β_4_ nAChR with *K_i_* values of 2.28 and 3.18 nM are high, almost the same as those of the lead compounds, **(*S*)**-**QND8**, **(*S*)**-**T2**, and AT-1001 (*K_i_* = 2.48, 2.25, and 2.60 nM, respectively) [46]. The selectivity of **AK3** over the α_7_ subtype is the highest with a 3069-fold higher affinity to the α_3_β_4_ in comparison to the α_7_ subtype, whereas for all compounds the selectivity for the α_4_β_2_ subtype is much lower with a range of 10–220-fold. Molecular docking of **AK1**, **AK3,** and lead compounds to α_3_β_4_ and α_7_ nAChR homology models were performed to explain the remarkably high selectivity profile of **AK3** to α_3_β_4_ over α_7_ nAChRs.

**Figure 3:**
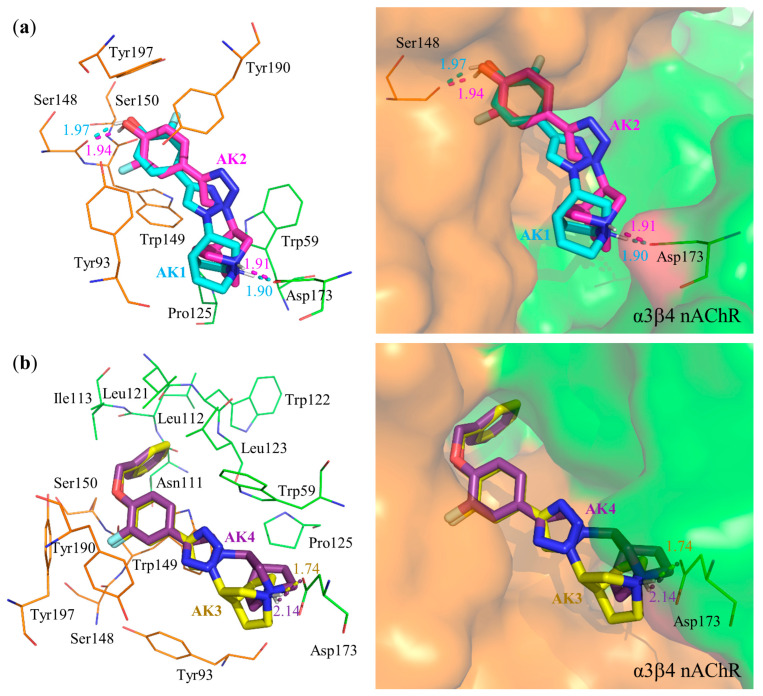
The overlay binding modes and molecular interactions of (*S*)- and (*R*)-enantiomers at the binding site of α_3_β_4_ nAChR: docked conformations of (**a**) **AK1** (cyan) and **AK2** (magenta) and (**b**) **AK3** (yellow) and **AK4** (violet). Orange and green indicated residues in α_3_-principal and β_4_-complementary subunits, respectively.

**Figure 4:**
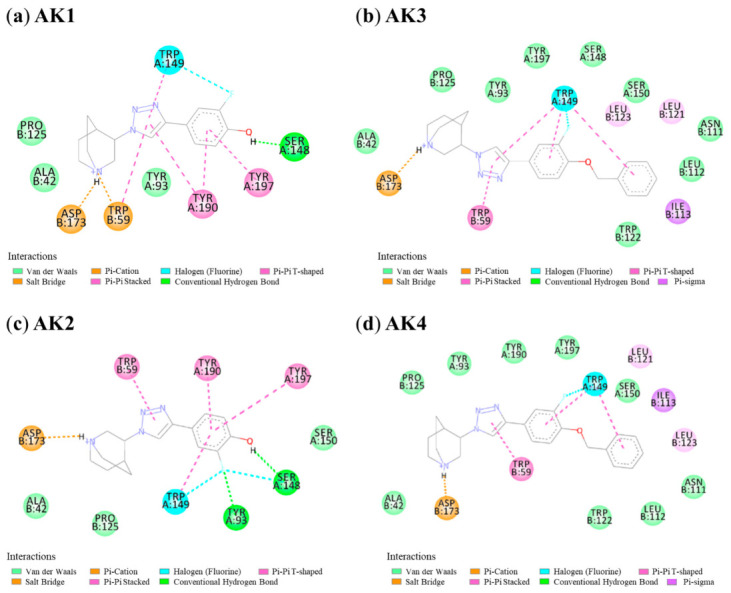
The binding interactions of (*S*)-enantiomers (**a**) **AK1** and (**b**) **AK3** and (*R*)-enantiomers (**c**) **AK2** and (**d**) **AK4** to α_3_β_4_ nAChR. A and B indicated α_3_ and β_4_ subunits, respectively.

The molecular docking of new compounds and lead compounds to α_3_β_4_ nAChR homology model showed that all compounds aligned and formed hydrogen bonds and π-π interactions with amino acid residues in an aromatic cage located at the interface between the α_3_ subunit and β_4_ subunit. The protonated quinuclidine ring of all (*S*)-enantiomers pointed to Asp173, a key amino acid determinant to form a salt bridge and hydrogen bond interaction in slightly shorter distances than their (*R*)-counterparts, particularly of 1.74 Å of **AK3** vs. 2.14 Å of **AK4** (Figure 3, Table 2). In agreement with a previous report [48], the (*S*)-enantiomer of a quinuclidine ring allowed the docked pose to accommodate a salt bridge interaction with Asp173, the key determinant of the α_3_β_4_ nAChR binding. Other common key residues of α_3_β_4_ nAChR for binding are Trp149 and Tyr190 in the α_3_ subunit for which the interactions can be observed to Trp149 and/or Tyr190 in the modified compounds. For **AK1**, the protonated quinuclidine formed a salt bridge interaction with Asp173, and the phenolic −O**H** formed a hydrogen bond with Ser148. The additional interactions were cation-π interaction with Trp59, π-π interactions with Trp59, Trp149, Trp190, and Tyr197, and halogen bond with Trp149 (Figure 4A and Table 2). Even though the number of hydrogen bonds and π-π interaction of **AK3** with key residues was less than those of **AK1**, the binding affinity of **AK3** to α_3_β_4_ nAChR (*K_i_* = 3.18 nM) was found to be comparable to **AK1** (*K_i_* = 2.28 nM). This result confirms that the extending benzyloxy group increased hydrophobic interactions as designed. These interactions from the expansion contributed to the similar binding affinity and ligand efficiency (LE), a parameter to assess the binding affinity of different molecular weights, of these two compounds (LE = −0.52 and −0.51 for **AK1** and **AK3**) (Table 2).

**Figure 5:**
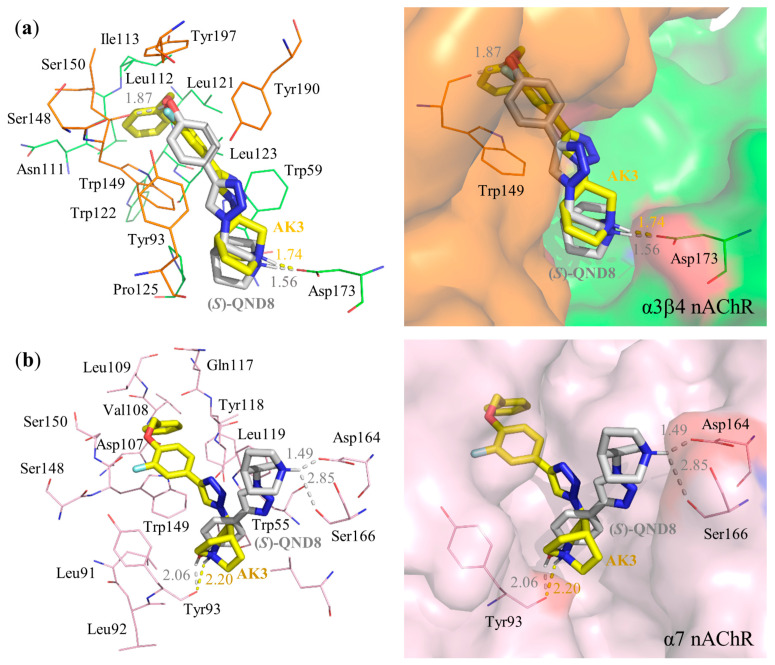
The overlay binding modes of **AK3** and **(*S*)**-**QND8** against (**a**) α_3_β_4_ nAChR and (**b**) α_7_ nAChR. Orange, green, and light pink indicated α_3_, β_4,_ and α_7_ subunits, respectively.

In terms of selectivity, **AK3** possesses a 3069-fold selectivity to α_3_β_4_ nAChR over α_7_ nAChR, whereas the selectivity of **AK1** and the lead compounds **(*S*)**-**QND8** and **(*S*)**-**T2** was much lower. The α_7_ nAChR binding poses of **AK3** and **(*S*)**-**QND8** showed that the quinuclidine ring of these two compounds is aligned in different directions leading to the higher number of hydrogen bond interactions of **(*S*)**-**QND8** (Figure 5B). Three hydrogen bond interactions of **(*S*)**-**QND8** to α_7_ nAChR are composed of the interaction of protonated quinuclidine to Asp164 and Ser166 and the interaction of -O**H** to Tyr93. Only one hydrogen bond interaction between protonated quinuclidine to Tyr93 was observed in **AK3** resulting in less preference for the α_7_ subtype. Hence, the extension approach, via the added benzyloxy group in the **AK3** structure, significantly enhanced the selectivity profile by the hydrophobic interactions with the residues in a β_4_-complementary subunit (Figure 3B). Therefore, the added benzyloxy group is the key contributor to the enhanced α_3_β_4_-selectivity of **AK3** by providing the hydrophobic interactions with the β_4_ subunit.

**Figure 6:**
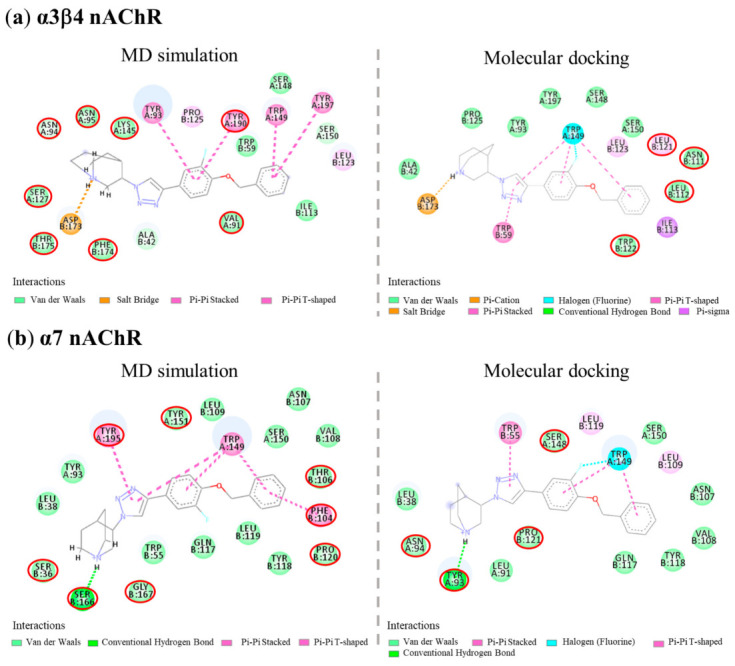
The binding modes of **AK3** to (**a**) α3β4 nAChR and (**b**) α7 nAChR from MD simulation compared to molecular docking. A and B indicate principal and complementary subunits, respectively. Red circles indicate different interacting amino acid residues between MD simulation and molecular docking.

MD simulation of **AK3** to α_3_β_4_ and α_7_ nAChRs was performed to gain more insight into the high affinity and selectivity of **AK3** to α_3_β_4_ nAChR (*K_i_* = 3.18 nM, 3069-fold selectivity over α_7_ nAChR). The strong interaction from the salt bridge formation between protonated quinuclidine and the carboxylate of Asp173, which appeared to mediate high affinity and selectivity to α_3_β_4_ nAChR, was detected by MD simulation. Besides the salt bridge formation, the major interactions observed in the MD simulation of **AK3** to α_3_β_4_ nAChR are four π-π interactions between both middle and terminal benzene rings of the hydrophobic part of **AK3** and residues Tyr93, Trp149, Tyr190, and Tyr 197 (Figure 6a). When compared with molecular docking, most of the interacting amino acid residues were the same (Tyr93, Ser148, Trp149, Ser150, and Tyr197 in principal α_3_ subunit and Ala42, Trp59, Ile113, Leu123, Pro125, and Asp173 in complementary β_4_ subunit) but the binding interaction types were altered, due to the flexible and solvated receptor in MD simulation. For example, the halogen bond between a fluorine atom and Trp149 and π-π interactions of a triazole ring to Trp59 and Trp149 observed in molecular docking were replaced by additional π-π interactions of middle and terminal benzene rings to Tyr93, Tyr190, and Tyr 197 in MD simulation (Figure 6a). Although the number of main interactions in the complexes of **AK3**-α_3_β_4_ nAChR and **AK3**-α_7_ nAChR, were equal (four π-π interactions), the salt bridge interaction between the protonated quinuclidine and Asp173 in **AK3**-α_3_β_4_ nAChR complex (Figure 6b) was stronger than the hydrogen bond interaction to Ser166 of α_7_ nAChR. The results from both molecular docking and MD simulation supported the higher affinity and selectivity of **AK3** to α_3_β_4_ nAChR than α_7_ nAChR.

**Figure 7:**
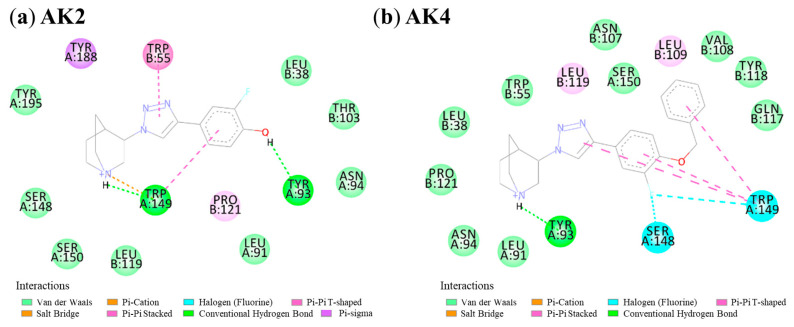
The binding interactions of (**a**) **AK2** and (**b**) **AK4** to α_7_ nAChR. A and B indicated principal and complementary subunits, respectively.

For (*R*)-enantiomers, **AK2** and **AK4** showed lower binding affinities to α_3_β_4_ nAChR with *K_i_* values of 601 and 112 nM, respectively than their (*S*)-counterparts and the lead compounds. From molecular docking results, the ligand-receptor interactions provided by quinuclidine and hydrophobic pharmacophores of **AK2** and **AK4** were not different from those of **AK1** and **AK3** (Figure 3 and Table 2). Only the triazole ring pointed to a different angle leading to the lower number of π-π interactions between the triazole ring of **AK2** and **AK4** with α_3_β_4_ nAChR compared to their (*S*)-counterparts (**AK1** and **AK3**) showing the important role of this moiety for the binding affinity (Figure 4). However, the (*R*)-enantiomers **AK2** and **AK4** bound to α_7_ nAChR with *K_i_* values of 4.49 and 53.6 nM, respectively, which are higher than the values of their (*S*)-counterparts (Table 1). The molecular docking to the α_7_ nAChR homology model showed that the quinuclidine ring interacted with Trp149 and Tyr93, key determinants of the α_7_ subtype leading to the high affinity of **AK2** and **AK4** (Figure 6). The key interactions of **AK2** are strong cation-π to Trp149, hydrogen bonds to Tyr93 and Trp149, and π-π interactions to Trp55 and Trp149, whereas **AK4** bound to the receptor with the hydrogen bond to Tyr93 and π-π interaction to Trp149 in addition to halogen bonds with Ser148 and Trp149 (Figure 7, Table 3). The absence of a strong cation-π interaction of the quinuclidine ring together with the lack of -OH to form additional hydrogen bonds resulted in the lower binding affinity of **AK4** compared to **AK2**. In addition, the extended benzyloxy group present in **AK4** might have caused steric hindrance to the receptor, leading to a decrease in affinity. The higher binding affinities of **AK2** than **AK4** (*K_i_* of 53.6 and 4.49 nM) were found to agree with the ligand efficiency (LE) of these two compounds: **AK4** bound to α_7_ nAChR weaker than **AK2** (LE = −0.47 and −0.52, respectively) (Table 3).

In terms of the structure-activity relationship (SAR) of quinuclidine-triazole derivatives targeting α_3_β_4_ nAChR, three components are required for high affinity and selectivity: the (*S*)-enantiomer of a quinuclidine ring, a triazole ring and a large hydrophobic group (extended benzene ring). Among four synthesized compounds, **AK3** showed the highest binding affinity and selectivity to α_3_β_4_ nAChR, qualifying this compound as a non-radioactive reference compound for α_3_β_4_ nAChR. Therefore, **AK3** is a high potential candidate for further development as α_3_β_4_ nAChR PET tracer for monitoring drug addiction, after replacing the fluorine atom with the radioactive isotope, fluorine-18.

## 3. Materials and Methods

### 3.1. Synthesis

All chemicals and solvents were purchased from Sigma-Aldrich (St. Louis, MO, USA), Merck (Darmstadt, Germany), AK Scientific (Union City, CA, USA), Oakwood (Estill, SC, USA), and Fisher Scientific (Waltham, MA, USA) and used without further purification. The NMR spectroscopic data (^1^H, ^13^C, COSY, HSQC, HMBC) were recorded with a Varian Mercury-300. High-Resolution Mass Spectra (HRMS) were recorded on the FT-ICR APEX II spectrometer using electrospray ionization (ESI) in positive ion mode.

**Scheme 1:**
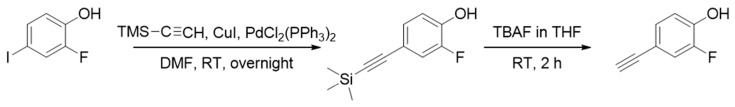
Sonogashira cross-coupling reaction and desilylation with TBAF in THF.

**Scheme 2:**
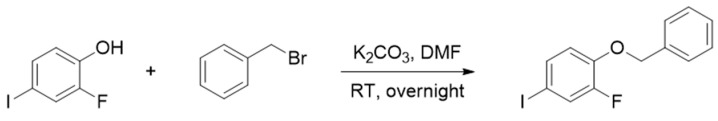
Nucleophilic substitution to prepare iodo-containing molecules.

**AK1**-**AK4** were synthesized by the previously described methods [47]. In brief, the terminal alkyne and quinuclidine azide were prepared first. For the alkyne building block, 2-fluoro-4-iodophenol reacted with trimethylsilyl acetylene in base condition using PdCl_2_(PPh_3_)_2_ and CuI as catalysts under nitrogen atmosphere overnight and purified by SiO_2_ column chromatography using 15%EtOAc in hexane as a mobile phase. The intermediate compound was desilylated with TBAF in THF at room temperature and purified by SiO_2_ column chromatography using 15%EtOAc in hexane as a mobile phase to get the terminal alkyne (Scheme 1) for **AK1** and **AK2**. For terminal alkyne of **AK3** and **AK4**, 2-fluoro-4-iodophenol first reacted with benzyl bromide in base condition at room temperature overnight (Scheme 2) before performing a Sonogashira cross-coupling reaction. The crude product was purified by SiO_2_ column chromatography using 5%Et_3_N, 10%MeOH in DCM as a mobile phase.

**Scheme 3:**
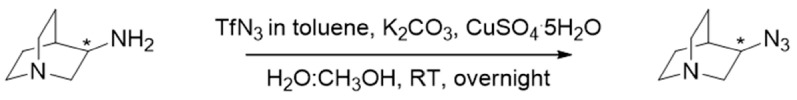
Azido quinuclidine preparation. * Indicates (*R*)- or (*S*)-enantiomer.

For the preparation of (*R*)- and (*S*)-quinuclidine azides, the reaction of trifluoromethanesulfonic anhydride and sodium azide was run in the mixture of water and toluene (1:1.5) at 0 °C for 2 h and the reaction mixture was extracted with toluene to yield trifluoromethanesulfonyl azide (TfN_3_). The freshly prepared TfN_3_ in toluene was added to (*R*)- or (*S*)- of 3-aminoquinuclidine, K_2_CO_3,_ and CuSO_4_.5H_2_O in the mixture of water and methanol (1:2) at room temperature overnight (Scheme 3). The prepared azide building blocks were then used without further purification.

**Scheme 4:**
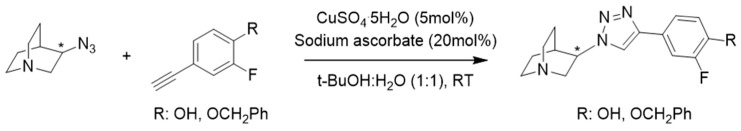
Copper-catalyzed azide-alkyne cycloaddition (CuAAC). * Indicates (*R*)- or (*S*)-enantiomer.

The prepared terminal alkynes and quinuclidine azides reacted via copper-catalyzed azide-alkyne cycloaddition (CuAAC) or click chemistry using 20 mol% of sodium ascorbate and 5 mol% of copper sulfate as catalysts to yield **AK1**-**AK4** as quinuclidine triazole compounds (Scheme 4).

**2-Fluoro-4-(1-((1*R*,3*S*,4*R*)-quinuclidin-3-yl)-1*H*-1,2,3-triazol-4-yl)phenol [AK1]**

(*S*)-3-Azidoquinuclidine (0.135 g, 0.91 mmol) reacted with 4-ethynyl-2-fluorophenol (0.170 g, 1.29 mmol) using 20 mol% of sodium ascorbate and 5 mol% of copper sulfate as catalysts to yield a white solid (70.90 mg, 71.43%). R_f_ = 0.25, MP: 258 °C (dec); FTIR (ATR) (cm^−1^): 3384 (O-H stretching), 2943, 2878 (aliphatic C-H stretching), 1619, 1566 (aromatic C=C stretching), 1459 (aliphatic C-H bending), 1293 (aromatic C-N stretching), 1200 (aliphatic C-N stretching), 1049 (aliphatic C-O stretching), 874, 781, (C=C bending); ^1^H NMR (300 MHz, DMSO-d_6_) δ 8.63 (s, 1H), 7.62 (dd, *J* = 12.4, 1.9 Hz, 1H), 7.52 (d, *J* = 7.90 Hz, 1H), 7.02 (m, 1H), 4.75 (m, 1H), 3.35 (m, 2H), 2.97 (m, 1H), 2.80 (m, 3H), 2.17 (m, 1H), 1.73 (m, 2H), 1.41 (m, 2H); ^13^C NMR (75 MHz, DMSO-d_6_) δ 153.24, 150.23, 145.95, 145.01, 122.04, 120.61, 118.58, 113.35, 58.22, 52.10, 46.94, 46.78, 27.96, 25.30, 19.82; HRMS (ESI) calculated (C_15_H_18_FN_4_O, MH^+^): 289.1459, found 289.1455.

**2-Fluoro-4-(1-((1*S*,3*R*,4*S*)-quinuclidin-3-yl)-1*H*-1,2,3-triazol-4-yl)phenol [AK2]**

(*R*)-3-Azidoquinuclidine (0.275 g, 1.81 mmol) reacted with 4-ethynyl-2-fluorophenol (0.351 g, 2.58 mmol) using 20 mol% of sodium ascorbate and 5 mol% of copper sulfate as catalysts to yield a white solid (143.2 mg, 27.62%). R_f_ = 0.25, MP: 275 °C (dec); FTIR (ATR) (cm^−1^): 3128 (O-H stretching), 2948, 2870 (aliphatic C-H stretching), 1619, 1563 (aromatic C=C stretching), 1462 (aliphatic C-H bending), 1293 (aromatic C-N stretching), 1200 (aliphatic C-N stretching), 1035 (aliphatic C-O stretching), 883, 781, (C=C bending); ^1^H NMR (300 MHz, DMSO-d_6_) δ 8.62 (s, 1H), 7.61 (dd, *J* = 12.4, 1.9 Hz, 1H), 7.53 (d, *J* = 7.90 Hz, 1H), 7.01 (m, 1H), 4.72 (m, 1H), 3.31 (m, 2H), 2.96 (m, 1H), 2.76 (m, 3H), 2.15 (m, 1H), 1.71 (m, 2H), 1.38 (m, 2H); ^13^C NMR (75 MHz, DMSO-d_6_) δ 153.25, 150.07, 145.95, 145.20, 121.95, 120.56, 118.61, 113.55, 57.81, 52.35, 47.03, 46.79, 28.12, 25.79, 20.08; HRMS (ESI) calculated (C_15_H_18_FN_4_O, MH^+^): 289.1459, found: 289.1457.

**(1*R*,3*S*,4*R*)-3-(4-(4-(Benzyloxy)-3-fluorophenyl)-1*H*-1,2,3-triazol-1-yl)quinuclidine [AK3]**

(*S*)-3-Azidoquinuclidine (0.090 g, 0.60 mmol) reacted with 1-(benzyloxy)-4-ethynyl-2-fluorobenzene (0.120 g, 0.53 mmol) using 20 mol% of sodium ascorbate and 5 mol% of copper sulfate as catalysts to yield a white solid (271.5 mg, 62.07%). R_f_ = 0.625, MP:173–175 °C; FTIR (ATR) (cm^−1^): 3100, 3020 (aromatic C-H stretching), 2934, 2867 (aliphatic C-H stretching), 1630, 1585, 1510 (aromatic C=C stretching), 1451 (aliphatic C-H bending), 1380 (aromatic C-N stretching), 1274 (aliphatic C-N stretching), 1133, 1026 (aliphatic C-O stretching), 880, 793 (C=C bending); ^1^H NMR (300 MHz, DMSO-d_6_) δ 8.70 (s, 1H), 7.72 (dd, *J* = 12.6, 1.5 Hz, 1H), 7.65 (d, *J* = 8.6 Hz, 1H), 7.47 (m, 1H), 7.47 (m, 1H), 7.42 (m, 2H), 7.35 (m, 2H), 5.22 (s, 1H), 4.76 (m, 1H), 3.61 (m, 2H), 2.94 (m, 2H), 2.21 (m, 1H), 1.77 (m, 2H), 1.44 (m, 2H); ^13^C NMR (75 MHz, DMSO-d_6_) δ 153.63, 150.40, 145.78, 145.64, 136.48, 128.54, 128.14, 127.93, 121.34, 120.61, 115.91, 112.73, 70.31, 57.40, 51.95, 46.60, 27.65, 25.31, 19.60; HRMS (ESI) calculated (C_22_H_23_FN_4_O, MH^+^): 379.1929, found: 379.1925.

**(1*S*,3*R*,4*S*)-3-(4-(4-(Benzyloxy)-3-fluorophenyl)-1*H*-1,2,3-triazol-1-yl)quinuclidine [AK4]**

(*R*)-3-Azidoquinuclidine (0.234 g, 1.54 mmol) reacted with 1-(benzyloxy)-4-ethynyl-2-fluorobenzene (0.349 g, 1.54 mmol) using 20 mol% of sodium ascorbate and 5 mol% of copper sulfate as catalysts to yield a white solid (285.1 mg, 73.53%). R_f_ = 0.625, MP:180–181 °C; FTIR (ATR) (cm^−1^): 3100, 3040 (aromatic C-H stretching), 2937, 2864 (aliphatic C-H stretching), 1630, 1512 (aromatic C=C stretching), 1456 (aliphatic C-H bending), 1380 (aromatic C-N stretching), 1276, 1223 (aliphatic C-N stretching), 1127, 1018 (aliphatic C-O stretching), 883, 740 (C=C bending); ^1^H NMR (300 MHz, DMSO-d_6_) δ 8.70 (s, 1H), 7.71 (dd, *J* = 12.8, 1.9 Hz, 1H), 7.65 (d, *J* = 8.65, 1H), 7.49 (m, 1H), 7.47 (m, 1H), 7.42 (m, 2H), 7.35 (m, 2H) 5.22 (s, 2H), 4.76 (1H), 3.61 (m, 2H), 2.94 (m, 4H), 2.21 (m, 1H), 1.77 (m, 2H), 1.44 (m, 2H); ^13^C NMR (75 MHz, DMSO-d_6_) δ 153.63, 150.40, 145.78, 145.11, 136.48, 128.54, 128.13, 121.96, 120.99, 112.99, 112.73, 70.31, 57.44, 51.97, 46.61, 27.67, 25.35, 19.63; HRMS (ESI) calculated (C_22_H_23_FN_4_O, MH^+^): 379.1929, found: 379.1927.

### 3.2. Binding Affinity

The binding affinities of quinuclidine triazole compounds **AK1**-**AK4** to nAChRs were determined by radioligand displacement assays [46]. SH-SY5Y cells stably transfected with human α_7_ nAChR and HEK293 cells stably transfected with human α_4_β_2_ nAChR or α_3_β_4_ nAChR were used in the experiments. Cells were collected, sedimented (800 rpm, 3 min), diluted with 50 mM TRIS-HCl, pH 7.4, and stored at −25 °C until use. Frozen cell suspensions were thawed and homogenized by a 27-gauge needle and diluted with incubation buffer (50 mM TRIS-HCl, pH 7.4, 120 mM NaCl, and 5 mM KCl). The membrane suspension was incubated with (±)-[^3^H]epibatidine (0.3 to 0.6 nM final concentration; molar activity 2.22 GBq/mmol). Nonspecific binding was determined by co-incubation with 300 µM (-)-nicotine tartrate. The incubation was performed at room temperature for 120 min and terminated by rapid filtration using Whatman GF/B glass-fiber filters presoaked in 0.3% polyethyleneimine and a 48-channel harvester (Biomedical Research and Development Laboratories, Gaithersburg, MD, USA) followed by 4 times washing with ice-cold 50 mM TRIS-HCl, pH 7.4. Filter-bound radioactivity was quantified by liquid scintillation counting. The 50% inhibition concentrations (IC_50_) were estimated from the competition curves by nonlinear regression using GraphPad Prism software and the *K_i_* values were calculated according to the Cheng-Prusoff equation [49].

### 3.3. Molecular Docking

The homology models of α_3_β_4_ and α_7_ nAChRs were prepared as described in our previous study [48]. Briefly, the human amino acid sequences of α_3_β_4_ and α_7_ nAChRs were downloaded from UniProt for searching a proper protein template from Protein Data Bank (PDB) by Blast protein in Chimera 1.10.2 (Resource for Biocomputing, Visualization, and Informatics at the University of California, San Francisco, CA, USA). The amino acid sequences of α_3_β_4_ nAChR or α_7_ nAChR and acetylcholine binding protein (AChBP) (PDB ID 5AFH) used as a template were aligned by Clustal Omega (Conway Institute, University College Dublin, Dublin, Ireland) and the homology model of α_3_β_4_ nAChR and α_7_ nAChR was generated by Modeller 9.15 (University of California San Francisco, San Francisco, CA, USA). Several parameters i.e., the Discrete Optimized Protein Energy (DOPE) score, the GA341 score, and the Ramachandran plot were used to evaluate model quality. The structures of **AK1**–**AK4** were drawn as protonated forms by Chem Draw Ultra 12.0 program (PerkinElmer, Waltham, MA, USA). The parameters for the molecular docking study with AutoDock4.2 included: 100 GA runs, a population size of 150, a maximum of 10,000,000 evaluations, and a maximum of 27,000 generations. The similar 3D conformations orientation within 2.0 Å were grouped as conformation clusters. The docked poses in the highest cluster as well as free binding energies (∆G binding) and ligand efficiency (LE) were analyzed. The binding interactions between ligand and target protein were visually analyzed by AutoDock4.2 in addition to BIOVIA Discovery Studio Visualized (Biovia, San Diego, CA, USA).

### 3.4. Molecular Dynamics (MD) Simulation

The molecular docking complexes of **AK3** to a homology model of α_3_β_4_ and α_7_ nAChRs were optimized by MD simulation using NAMD software (University of Illinois at Urbana-Champaign, Urbana, IL, USA) [50] with CHARMM force field [51]. The complexes were solvated in the TIP3P model water box. The charge of the system was neutralized with an appropriate number of counter ions. Initially, the water box was minimized by the conjugate gradient method. Before the MD simulation, the system was equilibrated for 200 ps using an NPT ensemble at 310 K and 1 atm which was controlled by the Nosé-Hoover Langevin piston method [52] with 2 fs time steps and SHAKE algorithm. Periodic boundary conditions (PBC) and Particle Mesh Ewald (PME) method [52] were used for calculation. In the production steps, 100 ns of MD simulations were performed with trajectories saving every 2 ns for analysis. The complexes’ stability was evaluated using root mean square deviation (RMSD) (Supplementary Figure S1). Finally, the complexes were analyzed by BIOVIA Discovery Studio 2020 (Biovia, San Diego, CA, USA) [53].

### 3.5. Statistical Analysis

Data are represented as mean derived from two independent experiments performed in triplicate. The mean differences were statistically analyzed by one-way ANOVA followed by Tukey’s multiple comparison test using GraphPad Prism software.

## 4. Conclusions

The structural modification of α_3_β_4_ nAChR ligands for the development of a PET tracer has been achieved. The newly designed quinuclidine-triazole derivative **AK3** showed good binding affinity (*K_i_* = 3.18 nM) and a significantly enhanced selectivity α_7_ nAChR (3069-fold). The structural features contributing to the significant improvement of affinity and selectivity profiles of **AK3** are the (*S*)-enantiomer of the quinuclidine ring, the triazole linker, and the extended hydrophobic part for interaction with the β_4_ subunit. The presence of a fluorine atom in **AK3** provides the opportunity to develop **AK3** as an α_3_β_4_ nAChR-targeted PET tracer. Therefore, **AK3** is a promising compound for further development as a drug-seeking behavior monitoring agent.

## Figures and Tables

**Table 1:** In vitro binding affinity constants (*K_i_* values) for binding of the quinuclidine-triazole derivatives towards human α_3_β_4_, α_4_β_2_, and α_7_ nAChRs*^a^*.

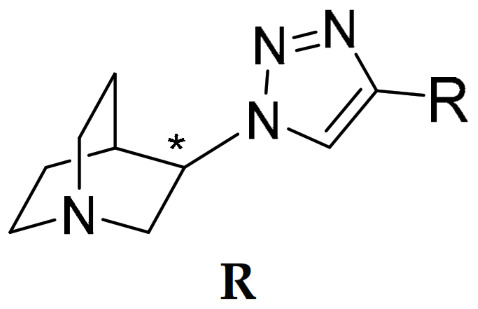 R	Binding Affinity *K_i_* (nM) *^a^*			Selectivity Ratios		
	α_3_β_4_ *^b^*	α_7_ *^c^*	α_4_β_2_ *^c^*	α_7_/α_3_β_4_	α_4_β_2_/α_3_β_4_	α_4_β_2_/α_7_
**Synthesized compounds**						
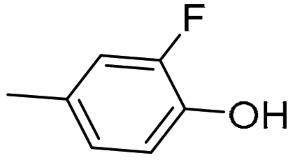 **AK1** (*S*-isomer)	2.28 (1.72; 2.85)	26.7 ^$^ (20.0; 33.4)	504 (196; 813)	11.7	221	18.9
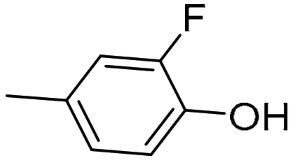 **AK2** (*R*-isomer)	601 ^#,$^ (432; 770)	4.49 ^$^ (3.56; 5.43)	5977 ^#,$^ (4939; 7015)	0.01	9.95	1331
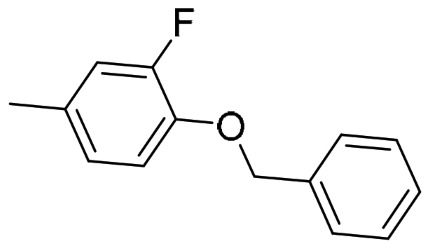 **AK3** (*S*-isomer)	3.18 (2.17; 4.18)	9760 ^#,$^ (9565; 9955)	180 (160; 201)	3069	56.6	0.02
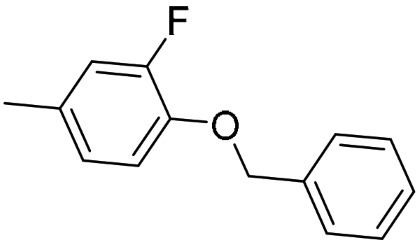 **AK4** (*R*-isomer)	112 (81.3; 142)	53.6 ^$^ (50.2; 57.1)	4176 ^#,$^ (4059; 4293)	0.48	37.3	77.9
**Lead compounds**						
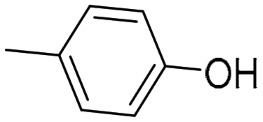 **(*S*)-QND8** [46]	2.48 ± 0.04	29.3 ± 0.18 ^$^	461 ± 89	11.8	186	15.7
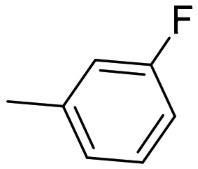 **(*S*)-T2** [46]	2.25 ± 0.42	660 ± 1.39 ^#^	519 ± 20	294	231	0.79

*^a^* Values represent the mean of two independent experiments performed in triplicates and reported in brackets. *^b^* Binding assays were performed with membrane preparations from HEK293 cells stably transfected with human α_3_β_4_ or α_4_β_2_ nAChR and the radiotracer [^3^H]epibatidine (working concentration ~ 0.5 nM; K_d_ = 0.025 nM for hα_4_β_2_ nAChR and K_d_ = 0.117 nM for hα_3_β_4_ nAChR). *^c^* Binding assays were performed with membrane preparations from SH-SY5Y cells stably transfected with human α_7_ nAChR and the radiotracer [^3^H]methyllycaconitine (working concentration ~ 0.5 nM; K_d_ = 2.0 nM). ^#^ *p* < 0.05 compared with (*S*)-QND8; ^$^ *p* < 0.05 compared with (*S*)-T2.

**Table 2:** The amino acid residues involved in the binding interaction to α_3_β_4_ nAChRs.

Cpds	α_3_β_4_ nAChRs				Binding Free Energy (ΔG, kcal/mol)	Ligand Efficiency * (LE)
	H-Bond (Distance in Å)	Cation-π	π-π	Halogen		
**AK1**	Ser148 (1.97), **Asp173** (1.90)	Asp173, Trp59	Trp59, Trp149, Tyr190, Tyr197	Trp149	−10.94	−0.52
**AK2**	Ser148 (1.94), **Asp173** (1.91)	Asp173	Trp59, Tyr190, Tyr197	Ser148, Trp149	−11.36	−0.54
**AK3**	**Asp173** (1.74)	Asp173	Trp59, Trp149	Trp149	−14.21	−0.51
**AK4**	**Asp173** (2.14)	Asp173	Trp59, Trp149	Trp149	−14.14	−0.51
**(*S*)**-**QND8**	Trp149 (1.87), **Asp173** (1.56)	Asp173	Tyr190	-	−13.28	−0.66
**(*S*)**-**T2**	**Asp173** (1.85)	Asp173	Trp59, Trp149, Tyr190, Tyr197	Ser148	−10.42	−0.52

The hydrogen bonds were analyzed and measured by AutoDock4.2, and the salt bridges, cation-π, and π-π interactions were analyzed by BIOVIA Discovery Studio Visualized. The interaction of amino acid residues with protonated quinuclidine is presented in bold. * LE is the ratio of Gibbs free energy (ΔG) to the number of non-hydrogen atoms of the ligand.

**Table 3:** The amino acid residues involved in the binding interaction to α_7_ nAChRs.

Cpds	α_7_ nAChRs				Binding Free Energy (ΔG, kcal/mol)	Ligand Efficiency * (LE)
	H-Bond (Distance in Å)	Cation-π	π-π	Halogen		
**AK1**	**Tyr93 ** (2.06, 2.03), **Ser148** (2.66)	Tyr195	Trp55, Trp149, Tyr188	-	−11.08	−0.53
**AK2**	Tyr93 (2.13), **Trp149** (1.86)	Trp149	Trp55, Trp149	-	−10.98	−0.52
**AK3**	**Tyr93 ** (2.20)	-	Trp55, Trp149	Trp149	−13.10	−0.47
**AK4**	**Tyr93** (2.15)	-	Trp149	Ser148, Trp149	−13.20	−0.47
**(*S*)**-**QND8**	Tyr93 (2.06), **Asp164** (1.49), **Ser166** (2.85)	-	Trp55	-	−11.18	−0.56
**(*S*)**-**T2**	-	Tyr55	Trp149, Tyr195	Ser148, Trp149	−9.99	−0.50

The hydrogen bonds were analyzed and measured by AutoDock4.2, and the salt bridges, cation-π, and π-π interactions were analyzed by BIOVIA Discovery Studio Visualized. The amino acid residues’ interaction with protonated quinuclidine is presented in bold. * LE is the ratio of Gibbs free energy (ΔG) to the number of non-hydrogen atoms of the ligand.

## Data Availability

All data underlying the results are available as part of the article and no additional source data are required.

## Supplementary Materials

The following supporting information can be downloaded at: https://www.mdpi.com/article/10.3390/ijms24043614/s1.
