# Supplementary material for: Selective α3β4 Nicotinic Acetylcholine Receptor Ligand as a Potential Tracer for Drug Addiction
Source: Int J Mol Sci. 2023 Feb 10;24(4):3614. doi: 10.3390/ijms24043614 (PMC9959096; doi:10.3390/ijms24043614)
Supplement: Supplementary file 1 [file ijms-24-03614-s001.zip › ijms-2047640-supplementary.pdf]

Supplemental information

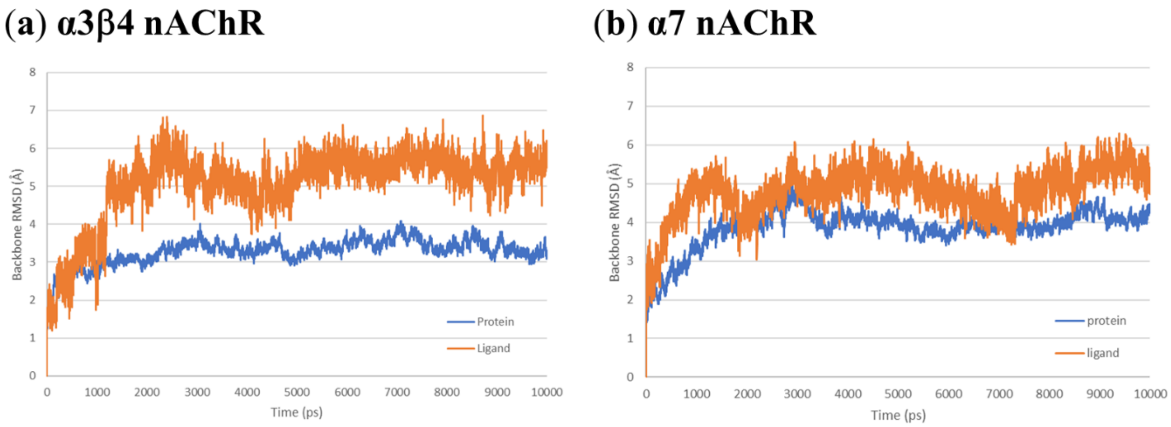

Figure S1. Root mean square deviation (RMSD) of **AK3** with (**a**) $\alpha_3\beta_4$ nAChR and (**b**) α_7 nAChR homology model from the initial conformation performed during molecular dynamic simulations.
